# Clinical characteristics of microbiologically inappropriate empiric antibiotics in emergency department patients with bacteremia: A descriptive study

**DOI:** 10.1097/MD.0000000000050882

**Published:** 2026-09-25

**Authors:** Akira Komori, Naohiro Kusunoki, Takako Kainoh, Katsuhiro Nagata

**Affiliations:** a Department of Emergency and Critical Care Medicine, Tsukuba Memorial Hospital, Tsukuba, Japan; b Department of General Medicine, Juntendo University Faculty of Medicine, Tokyo, Japan.

**Keywords:** antimicrobial resistance, antimicrobial stewardship, bacteremia, emergency department, sepsis

## Abstract

Septic patients frequently receive empiric antibiotics before microbiological results are available, and some regimens fail to cover the eventual causative pathogen. Such microbiologically inappropriate empiric therapy has been associated with worse clinical outcomes; however, the patterns and contributing circumstances leading to these mismatches in real-world emergency care remain insufficiently described. We conducted a single-center retrospective study of adult patients with bacteremia who received empiric intravenous antibiotics in a Japanese emergency department from January to December 2024. Microbiologically inappropriate empiric therapy was defined as the absence of in vitro susceptibility of the isolated bloodstream pathogen to the initial regimen. Clinical characteristics, infection source estimation, and pathogen profiles were examined descriptively. Among 118 patients with bacteremia who met inclusion criteria, 11 (9.3%) received microbiologically inappropriate empiric therapy. The median age was 82 years, and all patients met sepsis-3 criteria. Most mismatches were attributable either to infections caused by extended-spectrum β-lactamase–producing Enterobacterales despite correct initial source estimation or to inaccurate presumptive identification of the infectious source, which led to the selection of an ineffective empiric regimen. Mortality was more frequent among patients with incorrect initial source estimation. In conclusion, 2 distinct patterns contributed to inadequate empiric antibiotic coverage: resistant pathogens and diagnostic uncertainty regarding the infectious source. These findings suggest that antimicrobial stewardship efforts in emergency settings should not focus solely on antimicrobial spectrum selection but must also incorporate strategies to improve early source identification and timely recognition of resistant organisms at the point of care.

## 1. Introduction

Prompt initiation of appropriate empiric antibiotics is a key determinant of survival in sepsis.^[1]^ Clinicians must often initiate therapy before comprehensive assessment is completed, particularly in emergency departments (EDs) where time and resources for evaluating infection focus and resistance risks are limited. As a result, some patients receive empiric regimens that do not cover the eventual causative pathogen.^[2]^

Although numerous studies have examined the impact of inappropriate empiric therapy among hospitalized or intensive care unit patients,^[3–6]^ evidence specific to ED settings – where diagnostic uncertainty and time pressure are greatest – remains limited. Furthermore, prior ED-focused research has shown that misidentification of the infection source worsens prognosis;^[7]^ however, it has not evaluated antimicrobial susceptibility or the adequacy of initial empiric coverage. As a result, the mechanisms through which microbiological mismatches arise during early ED decision-making remain insufficiently characterized. Clarifying when and why empiric coverage fails in this high-stakes environment is essential for informing stewardship interventions and improving clinical outcomes.

To address this gap, we investigated the clinical characteristics of microbiologically inappropriate empiric antibiotic therapy among patients with bacteremia admitted through a Japanese ED.

## 2. Methods

### 2.1. Study design and setting

This single-center, retrospective, descriptive study was conducted at Tsukuba Memorial Hospital, Tsukuba, Japan. The hospital has 8 intensive care unit beds and provides 24-hour emergency services, with approximately 11,000 ED visits and about 6000 ambulance transports annually.

### 2.2. Patient selection

All patients who presented to the ED between January 1 and December 31, 2024, were screened. Patients were eligible if blood cultures were obtained, empiric intravenous antibiotics were initiated in the ED, they were admitted to the hospital, and subsequently at least 1 blood culture yielded a pathogen. Patients were excluded if the physicians judged their positive blood cultures to represent contamination.

### 2.3. Data collection

From electronic medical records, we abstracted demographic data, comorbidities relevant to the Charlson Comorbidity Index, and the level of long-term care dependency, which was classified into 7 grades (support levels 1–2 and care levels 1–5),^[8,9]^ as well as vital signs and laboratory findings at ED presentation. We also collected data on risk factors for drug-resistant organisms,^[10–12]^ including previous (within 3 months) antibiotic exposure, the presence of central venous or peripherally inserted central catheters, residence in a nursing facility, maintenance dialysis, previous detection of antimicrobial-resistant bacteria, and previous (within 3 months) hospitalization. In addition, information on immunosuppressive conditions (ongoing chemotherapy, immunosuppressant, or corticosteroid use), empiric antibiotic regimens, blood culture results including antimicrobial susceptibility, initial presumptive infectious focus, final confirmed diagnosis, admitting ward, and hospital disposition was collected.

### 2.4. Definitions

Empiric therapy was defined as microbiologically inappropriate if the pathogen isolated from blood cultures lacked in vitro susceptibility to the initial empiric antimicrobial regimen. The initial presumptive infectious focus and final diagnosis were based on the attending physicians’ chart documentation and independently verified by 2 emergency physicians, with disagreements resolved by consensus.

### 2.5. Statistical analysis

Data were summarized as medians with interquartile ranges (IQRs) for continuous variables and as counts with percentages for categorical variables. Given the small sample size, no inferential statistical tests were performed.

### 2.6. Ethics approval and consent to participate

This study protocol was approved by the Research Ethics Committee of the Tsukuba Memorial Hospital (IRB No. R07–03-02). Owing to the retrospective and anonymized nature of this study, the Research Ethics Committee waived the requirement for informed consent.

## 3. Results

During the 12-month study period, 163 adult patients were admitted with positive blood cultures. After applying the exclusion criteria, 118 patients constituted the final study cohort. Among these, 11 patients (9.3 %) received empiric antibiotic therapy that was microbiologically inappropriate. Detailed characteristics of these patients are presented in Table 1.

**Table 1:** Characteristics of 11 patients with bacteremia receiving microbiologically inappropriate empiric antibiotics.

No.	Age	Sex	Level of LTCD	Comorbidities	Risk factors for resistant organisms	SOFA score	Septic shock	Presumptive infectious site	Final diagnosis	Blood culture	Abx	Admission place	Disposition
1	82	W	C2	CVD	NA	3	No	Urine	UTI	ESBL-*E. coli*	CTRX	Ward	Home
2	82	W	C5	CHF PVD Dementia	pAbx Resident	8	No	Lung	STI	MRSA	T/P	ICU	Dead
3	66	M	C4	DM CVD	Recent Abx Resident pAMRB	10	Yes	Urine	UTI	ESBL-*E. coli*	T/P	ICU	Transfer
4	90	M	C4	DM CVD	NA	3	No	Urine	Enteritis	*C. perfringens*	CTRX	Ward	Dead
5	84	W	NA	DM	NA	3	No	Urine	UTI	ESBL-*E. coli*	CTRX	Ward	Home
6	86	M	C5	Malignancy CVD Dementia	pAbx Resident	5	No	Urine	CAUTI	ESBL-*E. coli*	T/P	Ward	Dead
7	90	W	C3	NA	Resident	7	No	Urine	CAUTI	ESBL-*E. coli*	CTRX	Ward	Home
8	77	M	S1	Malignancy	pAbx	3	No	Lung	SAB	MRSA	CTRX	Ward	Dead
9	77	W	C1	CHF	pAbx	2	No	Urine	UTI	ESBL-*E. coli*	CTRX	Ward	Home
10	83	M	C3	Malignancy CVD	NA	2	No	Lung	UTI	ESBL- *K. pneumoniae*	S/A	Ward	Transfer
11	71	W	N.A.	DM	NA	2	No	Bile duct	Cholangitis	*E. faecalis*	CMZ	Ward	Home

Level of long-term care dependency was classified into 7 grades (support levels 1–2 [S1–S2] and care levels 1–5 [C1–C5]).

Previous antibiotics were defined as any systemic antibiotic administration within 3 months before hospital admission.

Previous antimicrobial-resistant bacteria were defined as any isolation of antimicrobial-resistant bacteria within 3 months before hospital admission.

Abx = antibiotics, CAUTI = catheter-associated urinary tract infection, CHF = chronic heart failure, CMZ = cefmetazole, CTRX = ceftriaxone, CVD = cerebral vascular disease, DM = diabetes mellitus, ESBL = extended-spectrum β-lactamase–producing, ICU = intensive care unit, LTCD = long-term care dependency, M = man, MRSA = methicillin-resistant Staphylococcus aureus, NA = not applicable, pAbx = previous antibiotics, pAMRB = previous antimicrobial-resistant bacteria, PVD = peripheral vascular disease, S/A = sulbactam/ampicillin, SAB = *Staphylococcus aureus* bacteremia, SOFA = sequential organ failure assessment, STI = soft tissue infection, T/P = tazobactam/piperacillin, UTI = urinary tract infection, W = woman.

The median age was 82 years (IQR: 77–85), and 5 (45.5 %) were men. Nine patients were certified as requiring long-term care. The median sequential organ failure assessment score was 3 (IQR: 2.5–6.0), and all cases met the sepsis-3 definition of sepsis. None of the patients had immunosuppressive conditions.

The urinary tract was the most common site of infection (7 cases). Causative pathogens included 7 cases of extended-spectrum β-lactamase (ESBL)–producing Enterobacterales, 2 cases of methicillin-resistant *Staphylococcus aureus*, 1 case of *Enterococcus* species, and 1 case of *Clostridium perfringens*.

The initial presumptive infectious focus agreed with the final confirmed infection source in 7 of the 11 cases, of which 6 involved ESBL-producing Enterobacterales. Four cases had an incorrect presumptive focus. Of the 4 deaths observed in the inappropriate therapy group, 3 involved incorrect estimation of the initial infectious focus, and 1 involved infection by a resistant organism.

## 4. Discussion

This descriptive study found that approximately 10 % of septic patients with bacteremia admitted through the ED received empiric antibiotics that did not cover the causative pathogen. Importantly, 2 distinct and clinically meaningful patterns of mismatch were identified, each reflecting different challenges in the decision-making process for initial empiric therapy in emergency care.

First, mismatches occurred in patients infected with resistant organisms, most commonly ESBL–producing Enterobacterales, even when the initial infectious focus was correctly estimated. These cases illustrate the persistent dilemma of balancing adequate empiric coverage with antimicrobial stewardship principles. Current guidelines recommend carbapenems as first-line therapy for serious ESBL infections,^[12]^ but broadening empiric coverage for all suspected cases risks unnecessary carbapenem use and may accelerate antimicrobial resistance.^[12,13]^ In practice, clinicians often depend on bedside assessment of risk factors to guide coverage decisions; however, risk factors may be absent, underestimated, or difficult to ascertain rapidly in the ED. These findings suggest that ESBL-related empiric mismatches may not be fully avoidable with current clinical tools alone. Faster pathogen identification technologies, such as molecular rapid diagnostics,^[14,15]^ may eventually help refine early decision-making, though their implementation must carefully weigh cost, feasibility, and clinical impact.

Second, inaccurate estimation of the infectious focus itself resulted in inappropriate empiric therapy. Prior research has shown that misidentification of infection source in sepsis is associated with delayed appropriate treatment and increased mortality,^[7]^ highlighting the significance of early diagnostic accuracy. In our cohort, mortality was more frequently observed in cases with source misestimation than in those involving resistant organisms, suggesting that improving diagnostic precision may have a greater immediate clinical impact than simply expanding empiric antimicrobial spectra. Our findings reinforce this concern and highlight the need for stewardship interventions focused on improving identification of infectious sources at the point of care. Structured sepsis pathways^[16]^ might help reduce errors in source identification.

A novel aspect of our study is its detailed characterization of microbiologically inappropriate empiric therapy during initial ED management by integrating initial source estimation with microbiological susceptibility. Previous studies have largely focused on the association between inappropriate empiric therapy and clinical outcomes, often in large cohorts of hospitalized or critically ill patients.^[2–6]^ Although previous ED-based research has demonstrated the prognostic importance of accurate source identification,^[7]^ the clinical circumstances underlying microbiological mismatch during initial empiric treatment in the ED remain less well characterized. By examining individual cases in detail, we found that mismatch occurred not only when the infectious source was incorrectly estimated but also when the source was correctly estimated but the causative pathogen was resistant to the selected empiric regimen.

The key take-home message from our findings is that improving the appropriateness of empiric antibiotic therapy in the ED may require more than simply broadening antimicrobial coverage. Although incorrect source estimation remains an important cause of inappropriate empiric therapy, microbiological mismatch may occur even when the infectious source is correctly estimated because antimicrobial susceptibility is unknown at the time of initial treatment. This inherent uncertainty should be recognized when balancing adequate empiric coverage against antimicrobial stewardship in the ED.

We acknowledge some limitations. First, this was a single-center, retrospective study with a limited sample size, which restricts generalizability and precluded inferential statistical analysis. Second, assessment of the initial presumptive infection focus relied on chart documentation; despite independent consensus review, some degree of subjectivity cannot be excluded. Third, patients who were discharged home from the ED at their initial visit and whose blood cultures subsequently became positive were not included, potentially leading to underestimation of inappropriate empiric therapy. Finally, risk factors for resistant organisms were abstracted from available records and may have been incomplete, which could bias the evaluation of risk-based prescribing. However, this limitation also reflects real-world emergency settings, and we believe the findings offer valuable insights into actual clinical practice.

## 5. Conclusion

Approximately 10% of patients with bacteremia admitted through the ED received empiric antibiotics without in vitro activity against the causative pathogen. The mismatches arose mainly from infection with resistant pathogens, particularly ESBL-producing Enterobacterales, and from inaccurate initial source identification. These findings highlight the need for antimicrobial stewardship that combines early recognition of resistant pathogens with more accurate source estimation.

## Author contributions

**Conceptualization:** Akira Komori.

**Data curation:** Akira Komori.

**Formal analysis:** Akira Komori.

**Investigation:** Akira Komori, Takako Kainoh.

**Methodology:** Akira Komori.

**Validation:** Naohiro Kusunoki, Takako Kainoh, Katsuhiro Nagata.

**Supervision:** Katsuhiro Nagata.

**Writing – original draft:** Akira Komori.

**Writing – review and editing:** Takako Kainoh, Katsuhiro Nagata.
